# When the Unexpected Happens: Uterine Rupture in an Unscarred Uterus

**DOI:** 10.7759/cureus.90794

**Published:** 2025-08-23

**Authors:** Asmita Kaundal, Poojan Marwaha, Bhavna Bhavna, G. Sathiya Kaniga

**Affiliations:** 1 Obstetrics and Gynaecology, All India Institute of Medical Sciences, Bilaspur, Bilaspur, IND

**Keywords:** maternal mortality, neonatal mortality, pregnancy, spontaneous uterine rupture, uterine rupture

## Abstract

Uterine rupture in an unscarred uterus is extremely rare, but when it occurs, it can lead to the worst maternal and neonatal outcomes due to delayed diagnosis and management. A high index of suspicion is required due to variable presentation. We present here a case of a multiparous woman at term with a spontaneous onset of labor and progression to the second stage. However, when she did not deliver even after two hours, she was referred to our center with a non-reassuring fetal heart rate. After the initial evaluation of the patient, a provisional diagnosis of uterine rupture with fetal demise was made. Emergency laparotomy was performed. A single dead fetus weighing 2.9 kg was extracted along with the placenta from the abdominal cavity. Uterine repair and bilateral tubal ligation were done. The patient was discharged on the 10th postoperative day in satisfactory condition.

## Introduction

Uterine rupture is the separation of all three layers of the uterus. Rupture of the uterus is a rare but catastrophic obstetric emergency endangering both the mother's and the unborn child’s lives. Uterine rupture has been commonly encountered during the delivery of women with a scarred uterus (previous cesarean section, previous myomectomy, repeated dilatation, and curettage, etc.), with an incidence of around 22 per 10,000 deliveries [1-2]. However, uterine rupture is also seen in an unscarred uterus, although the incidence is very low (0.2 per 10,000) [3]. Any factor that leads to myometrial weakness or exposes it to excessive stress can lead to uterine rupture. Overdistended uterus (multiple pregnancy, polyhydramnios, large for gestation age baby), obstetrics maneuvers (external cephalic version or internal podalic version, instrumental delivery), trauma ( direct trauma to gravid uterus or fundal pressure), multiparity, injudcious use of uterotonics, prolonged and neglected labor leading to obstructed labour are some of the known causes of rupture in an unscarred uterus. Certain genetic conditions, like Ehlers-Danlos syndrome, which is a connective tissue disorder associated with fragile blood vessels and hollow organs like the uterus, can also predispose the gravid uterus to rupture during pregnancy and labour. While most uterine ruptures occur during labor, a few may occur pre-labor without many warning signs or symptoms and hence are termed as silent rupture [3-4]. Stillbirth, obstetric hemorrhage, massive blood transfusion, obstetric hysterectomy, bladder injuries, obstetric fistula, and neonatal and maternal mortality are some of the known complications of a uterus rupture [5].

We present the case of uterine rupture in a multiparous antenatal woman with no prior history of uterine surgery or dilation and curettage (D&C), at term gestation, who went into spontaneous labour and progressed well into the second stage of labor without any augmentation. This report aims to highlight the clinical presentation, diagnosis, and surgical management of uterine rupture in an unscarred uterus, with the emphasis on meticulous monitoring of labor and early diagnosis and timely management, even in the absence of a history of any uterine surgery. 

## Case presentation

A 27-year-old G5P2L2A2 at 38 weeks+6 days of gestation was referred from a peripheral center in the second stage of labor with fetal distress due to the unavailability of an operating theater. She had two previous full-term normal vaginal deliveries and two first-trimester spontaneous abortions not requiring any surgical interventions. She had no significant past medical or surgical history. She was a migrant laborer. Her present pregnancy was unbooked and unsupervised. She had no previous antenatal investigations or ultrasound. According to her attendants, she started having labor pains at 8:00 am on the day of her presentation. She was brought to the peripheral center, where she was observed for the spontaneous progress of labor. She was shifted to the labor room at 4:00 pm, after two hours of trial for normal delivery, she was referred to a higher center for a non-reassuring fetal heart rate and the unavailability of an operating theater. There was no documentation of a uterotonic used for labour augmentation on the referral slip. She gives no history of instrumentation or fundal pressure. 

On presentation, the patient looked exhausted and distressed. She was mildly dehydrated, with a pulse rate of 126 beats per minute, blood pressure of 100/60 mmHg, a respiratory rate of 22 breaths/minute, a temperature of 37 °C, and an oxygen saturation of 99% on room air. Upon abdominal examination, her abdomen was distended, and tenderness was present all over the abdomen. Uterine contour could not be made out. The patient was complaining of constant pain, but no uterine contractions could be appreciated. The fetal heart was not audible with the stethoscope or Doppler. On per vaginal examination, her vagina was dry and hot, the cervix was fully dilated, the vertex was high up, and no liquor drained. Based on clinical examination, a provisional diagnosis of uterine rupture with intrauterine fetal demise was made. Because of the condition of the mother, confirmation of uterine rupture and fetal demise was not done by ultrasound, and, along with resuscitative measures, a decision for emergency exploratory laparotomy was taken. Her hemoglobin was 6.5 gm/dl, total leucocyte count was 25,000/microlitre, and platelet count was 2.5 lakhs (Table 1). Patients and relatives were informed regarding the maternal and fetal status, the need for emergency surgery, and risks, including the need for peripartum hysterectomy.

**Table 1:** Laboratory test results of the patient This table provides the details of the laboratory investigations of the patient, including the test names, unit of expression, patient results, and their biological reference values. gm/dl: gram/deciliter, mg/dl: milligram/deciliter, U/L: units/liter

Test	Preoperative patient investigations	Postoperative patient investigations	Units	Biological reference range
Complete blood count (CBC)				
Haemoglobin (Hb)	6.5	9.0	gm/dl	13.0-17.0
Total leucocyte count (TLC)	25.0	8.0	thousand/micro litre	4-10
Platelet count	250	1.89	thousand/micro litre	150-400
Random blood sugar	92		mg/dl	70-139
Liver function test				
Serum bilirubin	0.8		mg/dl	0.3-1.2
Alanine transaminase (ALT)	23		U/L	0-35
Aspartate aminotransferase (AST)	23		U/L	0-45
Alkaline phosphatase (ALP)	112		U/L	45-117
Kidney function test				
Blood urea	36		mg/dl	13-43
Blood urea nitrogen	17		mg/dl	8-20
Serum creatinine	0.8		mg/dl	0.90-1.30

**Figure 1:**
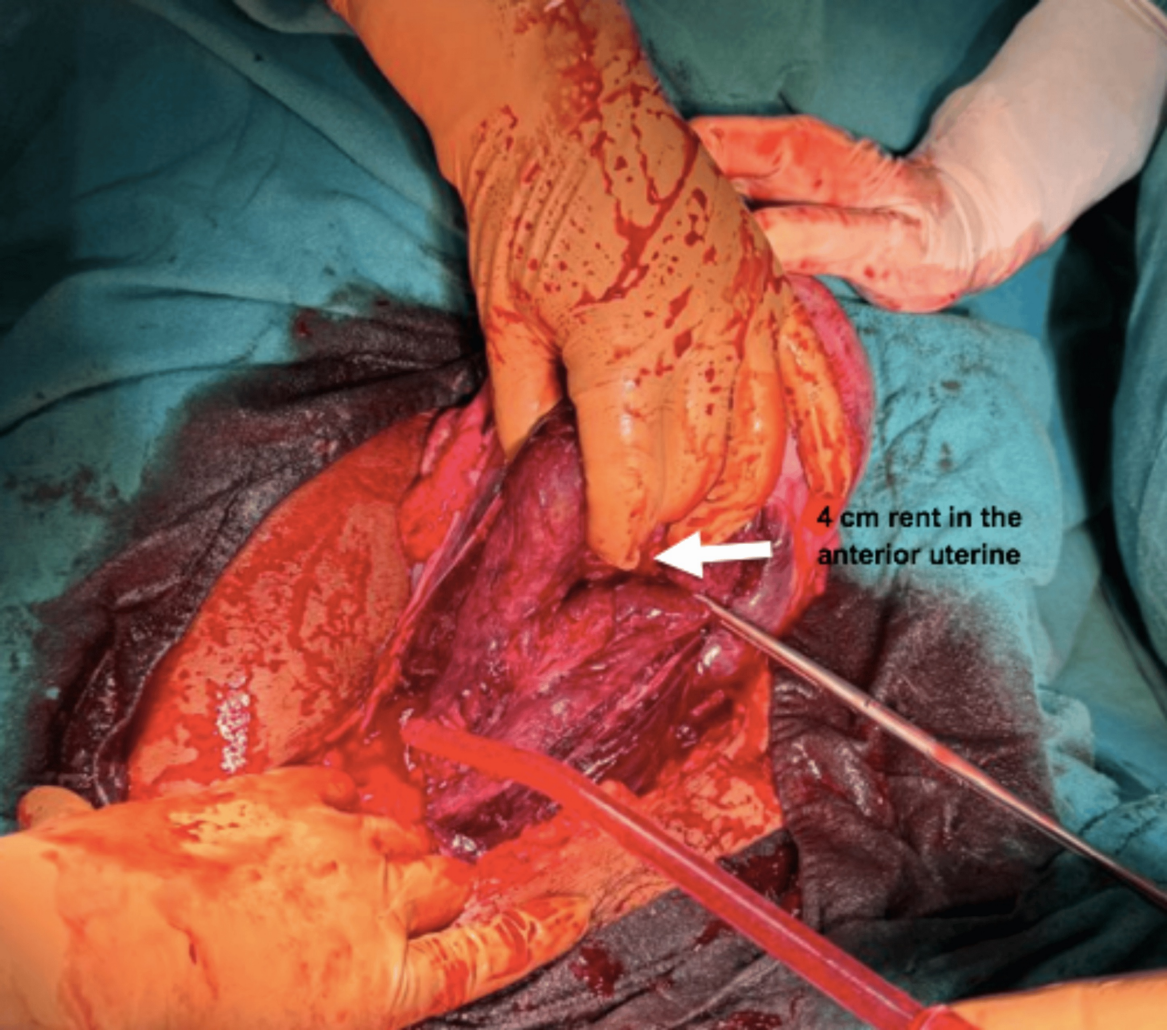
Anterior uterine wall rupture A 4 cm rent in the anterior uterine wall of the uterus (white arrow), located near the lower uterine segment, consistent with anterior uterine wall rupture.

**Figure 2:**
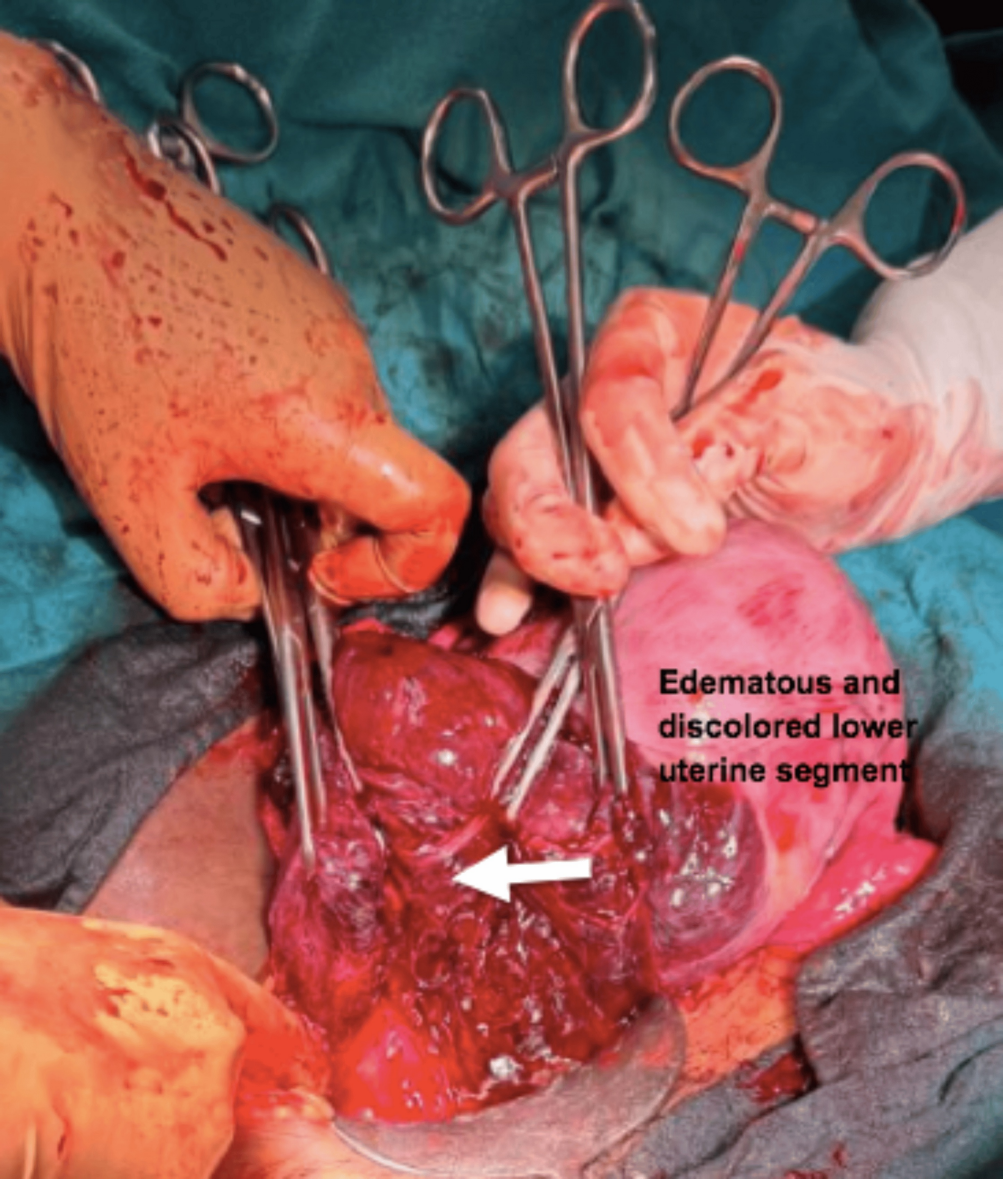
Edematous and discolored lower uterine segment and bladder An intraoperative image showing the edematous lower uterine segment with bluish discoloration of the lower uterine segment and bladder (white arrow), possibly due to congestion and ischemia due to prolonged pressure and obstructed labor subsequently leading to uterine rupture.

Intraoperatively, around two liters of hemoperitoneum were present. The baby and placenta were lying in the abdominal cavity. A fresh, dead baby weighing 2.9 kg was extracted along with the placenta. On inspection, around 4-cm-long rent was present, starting from the left lateral uterine wall to the anterior uterine wall. The lower uterine segment was deep blue to blackish. The rest of the uterus, bilateral ovaries, and fallopian tubes were normal in morphology. The bladder was oedematous (Figures 1, 2). Careful repair of the uterus was done in two layers. Bilateral tubal ligation was done. Hemostasis was achieved. Abdominal lavage was done. Intrabdominal drain inserted. The abdomen was closed in layers. The patient received three units of blood intraoperatively and one postoperatively. Breastmilk suppression was given. Her postoperative period was uneventful. She was discharged on day 10 of surgery.

## Discussion

Loss of integrity of all the layers of the uterus is known as uterine rupture. Although rare, it is the most severe obstetric complication and a nightmare for both healthcare providers and the antenatal woman. Uterine ruptures are more commonly seen in women with scarred uteri; they can occur in the unscarred uteri as well (22 vs. 0.2 cases per 10,000 deliveries) [1-3].

Multiparity, induced labor, inadvertent use of oxytocin, congenital abnormalities of the uterus, connective tissue disorders like Ehlers-Danlos syndrome, history of curettage, instrumental delivery, obstetric maneuvers, direct trauma, or fundal pressure are some of the important risk factors for rupture in an unscarred uterus [6]. Our patient is multigravida with previous two full-term births, making her more prone to uterine rupture. She had two spontaneous first-trimester abortions for which no surgical intervention was required. Although multiparity is a known risk factor for uterine rupture, there are case reports where uterine rupture has been seen in primigravida also [7-8].

Due to the diversity in presentation, early diagnosis is often challenging. Uterine rupture can occur at any time before the establishment of labor, in active labor, or during the second stage. Uterine rupture can also be diagnosed after delivery when the patient either suffers from intractable postpartum hemorrhage unresponsive to standard medical management or persistent pelvic pain, hemoperitoneum, and hemodynamic instability [9-10]. According to the WHO Labour Care Guide for a multiparous woman, the active first stage should not exceed 10 hours, and the second stage should not be allowed to exceed two hours. Our patient went into spontaneous labour and had a labour of more than eight hours, which included two hours of second-stage labour, after which she was referred for a non-reassuring fetal heart. Hence, it is clear that in our patient, birth could not be achieved within two hours of being in the second stage. Prolonged second stage is an important risk factor as it can lead to obstructed labour, and if cesarean delivery is not done in time, it can result in uterine rupture. Hence, timely referral to a higher center with facilities for maternal and fetal resuscitation (adult and neonatal intensive care units) and an operative facility is of utmost importance. Sudden severe abdominal pain, vaginal bleeding, loss of fetal movement, cessation of uterine contractions, vomiting, and abnormal fetal heart rate pattern can suggest the diagnosis of uterine rupture. On examination, signs of maternal exhaustion, tachycardia, hypotension, dyspnea, restlessness, dehydration, fever, abdominal distension, tenderness on palpation, difficulty making out the uterine contour, inaudible fetal heart, loss of fetal station on vaginal examination, hot and dry vagina may be seen [11].

Our patient was referred for a non-reassuring fetal heart, an early sign of uterine rupture. When she reached the facility, her uterine contractions had already ceased. Uterine contour could not be made out, abdomen was distended and tender. The fetal heart was not audible. All these features were consistent with uterine rupture. Ultrasound can help in the confirmation of diagnosis, where one can find an empty enlarged uterus, with fetus in the abdominal cavity, mostly with absent heart and presence of hemoperitoneum. Exploratory laparotomy with either uterine repair or abdominal hysterectomy is the definitive management. Uterine rupture is the most dreadful complication of pregnancy and labour and a direct cause of maternal and neonatal mortality. A high index of suspicion is required for early diagnosis and timely management to avoid adverse feto-maternal outcomes. Multiparous women, even without a history of prior uterine surgery, should be carefully monitored during labour and should be timely referred to a higher center. 

## Conclusions

Uterine rupture is one of the important causes of direct maternal deaths and an indicator of the quality of antenatal and delivery services. Risk stratification, timely referral of high-risk cases to higher centers for delivery, a high index of suspicion, meticulous monitoring of labor, and timely decision on cesarean section are crucial steps toward the prevention of these catastrophic results of uterine rupture. More meticulous labor monitoring in multiparous women and timely referral to higher centers, even in the absence of any history of previous uterine surgery, should be considered. Auditing the cases of uterine rupture is necessary for a better understanding of the lacunae in care and helps in formulating regional guidelines for the prevention of such events.
